# Supplementary material for: Observational Cross-Sectional Study on Mediterranean Diet and Sperm Parameters
Source: Nutrients. 2023 Dec 1;15(23):4989. doi: 10.3390/nu15234989 (PMC10707842; doi:10.3390/nu15234989)
Supplement: Supplementary file 1 [file nutrients-15-04989-s001.zip › nutrients-2693736-supplementary.pdf]

Supplemental Table S1

Correlation analysis between MEDAS Score and semen parameters, in 300 study participants, corrected for subjects age, body mass index and smoking status. Values in bold are significative.

Parameters		
Semen Volume	R	0.151
	P value	**0.009**
Semen pH	R	-0.036
	P value	0.538
Sperm Concentration	R	0.483
	P value	**<0.0001**
Total Sperm Count	R	0.545
	P value	**<0.0001**
Sperm Progressive Motility	R	0.524
	P value	**<0.0001**
Non-Motile Sperm	R	-0.500
	P value	**<0.0001**
Sperm Viability	R	0.425
	P value	**<0.0001**
Typical Sperm Morphology	R	0.538
	P value	**<0.0001**

MEDAS Questionnaire

Questions	*Answers*
1) Is olive oil the main culinary fat used?	Yes = 1 point / No = 0 point
2) Are ≥4 tablespoons of olive oil used each day?	Yes = 1 point / No = 0 point
3) Are ≥2 servings (of 200 g each) of vegetables eaten each day?	Yes = 1 point / No = 0 point
4) Are ≥3 servings of fruit (of 80 g each) eaten each day?	Yes = 1 point / No = 0 point
5) Is <1 serving (100–150 g) of red meat/hamburgers/other meat products eaten each day?	Yes = 1 point / No = 0 point
6) Is <1 serving (12 g) of butter, margarine or cream eaten each day?	Yes = 1 point / No = 0 point
7) Is <1 serving (330 mL) of sweet or sugar sweetened carbonated beverages consumed each day?	Yes = 1 point / No = 0 point
8) Are ≥3 glasses (of 125 mL) of wine consumed each week?	Yes = 1 point / No = 0 point
9) Are ≥3 servings (of 150 g) of legumes consumed each week?	Yes = 1 point / No = 0 point
10) Are ≥3 servings of fish (100–150 g) or seafood (200 g) eaten each week?	Yes = 1 point / No = 0 point
11) Is <3 servings of commercial sweets/pastries eaten each week?	Yes = 1 point / No = 0 point
12) Is ≥1 serving (of 30 g) of nuts consumed each week?	Yes = 1 point / No = 0 point
13) Is chicken, turkey, or rabbit routinely eaten instead of veal, pork, hamburger, or sausage?	Yes = 1 point / No = 0 point
14) Are pasta, vegetable or rice dishes flavored with garlic, tomato, leek or onion eaten ≥ twice a week?	Yes = 1 point / No = 0 point
